# crcTRP: A Translational Research Platform for Colorectal Cancer

**DOI:** 10.1155/2013/930362

**Published:** 2013-01-29

**Authors:** Ning Deng, Ling Zheng, Fang Liu, Li Wang, Huilong Duan

**Affiliations:** Department of Biomedical Engineering, Key Laboratory for Biomedical Engineering of Ministry of Education, Zhejiang University, Hangzhou 310027, China

## Abstract

Colorectal cancer is a leading cause of cancer mortality in both developed and developing countries. Transforming basic research results into clinical practice is one of the key tasks of translational research, which will greatly improve the diagnosis and treatments of colorectal cancer. In this paper, a translational research platform for colorectal cancer, named crcTRP, is introduced. crcTRP serves the colorectal cancer translational research by providing various types of biomedical information related with colorectal cancer to the community. The information, including clinical data, epidemiology data, individual omics data, and public omics data, was collected through a multisource biomedical information collection solution and then integrated in a clinic-omics database, which was constructed with EAV-ER model for flexibility and efficiency. A preliminary exploration of conducting translational research on crcTRP was implemented and worked out a set of clinic-genomic relations, linking clinical data with genomic data. These relations have also been applied to crcTRP to make it more conductive for cancer translational research.

## 1. Introduction

Nowadays, cancer is still one of the major diseases that endanger human life. As American Cancer Society reported, a total of 1,638,910 new cancer cases and 577,190 deaths from cancer were projected to occur in the United States in 2012 [1]. Among all kinds of cancer, colorectal cancer is the second leading cause of cancer death in the United States and the fifth leading cause in China [2, 3]. Though researches focusing on the molecular mechanism of colorectal cancer have made great progress, effective clinical measurements for early diagnoses and treatments are still very scarce due to the wide gap between laboratory research and clinical practice. Therefore, it is of great significance to conduct translational research, which can promote the transforming of basic research findings on colorectal cancer into clinical practice [4], for reducing the mortality of colorectal cancer.

Obtaining required data efficiently and conveniently is a necessity for the smooth conduct of translational research. However, biomedical data are usually stored in heterogeneous databases with different terminologies, since they are generated in several domains including clinical practice, epidemiology survey, and laboratory research by different institutions. Organizing and managing various types of biomedical data efficiently and then sharing these data through a public platform can help researchers surmount difficulties of data acquisition, which will contribute a lot to colorectal cancer translational research. 

In this study, we developed a translational research platform, named crcTRP, aiming at accelerating translational research for colorectal cancer. crcTRP is consisted of a multisource biomedical information collection solution, a clinic-omics database, and a web portal. The multisource biomedical information collection solution focuses on acquiring different types of biomedical information related with colorectal cancer from different data sources. The clinic-omics database is developed for integrating various types of data obtained through the information collection solution reasonably and effectively. While the web portal aims at sharing the whole information we acquired among physicians, molecular biologists, and other researchers related with colorectal cancer translational research. Using information provided by crcTRP, we then generated a set of clinic-genomic relations based on UMLS [5], which is a typical example for illustrating what contributions can be made by crcTRP in translational research. These relations were then been applied to crcTRP to make it better for translational research.

## 2. Methods

First, a multisource biomedical information collection solution was proposed to collect various biomedical data related to colorectal cancer, including clinical data, epidemiology data, individual omics data, and public omics data. Second, a clinic-omics database was constructed to organize and manage the obtained dataset. Third, a web portal was designed to share the obtained information among various researchers related with colorectal cancer translational research. Finally, we proposed a data mining method to map clinic-genomic relations from crcTRP.

### 2.1. Acquisition of Multisource Biomedical Information

The acquisition of multisource biomedical information includes clinical information collection based on SNOMED CT [6], epidemiology data collection based on in-house web application, individual omics data collection based on MIAME [7], and public biomedical data collection based on OMIM [8]. 

#### 2.1.1. Clinical Data Acquisition Based on SNOMED CT

With the rapid progress of health information technology, Electronic Medical Records systems (EMR systems) have been widely used in the hospitals. This way, it is possible for us to collect clinical data electronically. We designed a data acquisition interface to collect deidentified clinical data from these EMR systems. Private information such as name, date of birth, and medical record ID is removed so that the patient cannot be directly identified. The same clinical concept may have different expressions in different information systems, which will reduce the efficiency of data utilization. So we coded the clinical information standardizedly using SNOMED CT. As a compositional concept system, SNOMED CT provides a compositional syntax for building new biomedical concepts. This feature makes SNOMED CT present complex clinical concepts about colorectal cancer appropriately. Concepts already included in SNOMED CT were precoordinated, while concepts not included were postcoordinated. For example, “severe pain in the left abdomen” cannot be expressed using concepts in SNOMED CT directly, but it can be postcoordinated as:

  21522001 *|*abdominal pain*|*:
  272741003 *|*laterality*|* = 77710000 *|*left*|*,
  246113005 *|*severity*|* = 24484000 *|*severe*|*.

#### 2.1.2. Epidemiology Data Acquisition Based on In-House Web Application

Epidemiology data can help us gain a comprehensive understanding of high-risk population's dietary habits, personal medical history, family disease history and other factors which may cause the disease. We developed a web-based epidemiology data acquisition system for colorectal cancer. Designed with Browser/Server architecture, this system can be loaded to several mobile platforms. Moreover, by using advanced features of HTML5 including novel client-side storage method and offline web applications [9], this system can work on mobile devices smoothly even in places without network connection. Once the system has been loaded to a mobile device, researchers can collect epidemiology data using the mobile device at bedsides, communities, or other areas with highincidence of colorectal cancer (usually remote mountain areas) without concern about the internet connection.

#### 2.1.3. Individual Omics Data Acquisition Based on MIAME

Since translational informatics is ready to revolutionize human health and healthcare using large-scale measurements on individuals [10], massive individual omics data will emerge as expected. Biospecimens are the physical sources of individual omics data. Well-annotated biospecimens will aid scientists to validate their research and correlate their findings with the associated pathological and clinical annotations. Collecting specimen through a relatively standardized procedure can obtain more useful and valid annotation information. Therefore, we developed an information managing system for biospecimen and individual omics data to collect personalized genomic data as well as their annotation information. Our management procedure for specimens was inspired by caTissue [11], a biospecimen informatics system of caBIG [12], while the collected individual omics data were designed to satisfy the MIAME guidelines, which outline the minimum information about a microarray experiment. 

#### 2.1.4. Public Biomedical Data Acquisition Based on OMIM

With the rapid development of high throughput technology, massive amounts of molecular biology data such as nucleic acid data and protein data have emerged. Moreover, owing to the sharing culture in this field, data are now increasingly publicly available [13]. However, molecular biology data in different types are often stored in different database, which makes it inconvenient for researchers to utilize these data comprehensively. We proposed a scheme for extracting information about colorectal cancer from these databases through OMIM. OMIM, a comprehensive, authoritative compendium of human genes and genetic phenotypes, is freely available and updated frequently. First, we searched for genes related to colorectal cancer using the keywords combination “(colon or colorectal or colonic or rectal) and (cancer or carcinoma or adenoma)” in OMIM. We limited the search fields as title and allelic variants, considering that content provided by title and allelic variants field associates with the corresponding theme most closely [14]. Second, we searched for protein IDs and article IDs using the obtained gene IDs in files provided by NCBI [15], which are about the associations between genes and other biomedical data. Finally, we extracted gene, protein, and literature information related to colorectal cancer from public biomedical databases including NCBI Gene [16], RefSeq [17], PubMed [18], and Swiss-Prot [19] using IDs obtained in the first two steps.

### 2.2. Construction of a Clinic-Omics Database

Data collected through the multisource biomedical information collection solution can be categorized into individual information and public information. Individual information means information centering on patients, including clinical data, epidemiology data, and individual omics data. Public information refers to information collected from pubic biomedical database, including gene, protein, and literature information. Public information centers on omics data.

To manage all the data in a database efficiently, we analyzed the characteristics of these data. With the rapid development of molecular biology, new attributes of biomarkers or new biomarkers will be found undoubtedly, which results in the dynamic nature of individual omics data. Meanwhile, it is impossible for a certain patient to have all the items of clinical data, suggesting that clinical data such as symptoms and examinations of colorectal cancer are sparse. For example, not all patients need an MRI examination, causing the corresponding data item recording the test to result in the conventional database being null in most cases. On the contrary, epidemiology data and public information we acquired are relatively stable. Traditional ER model is not suitable for the dynamic and sparse nature of clinical omics data because of the fixed database schema. The EAV model, recording data based on entity-attribute-value, is more flexible and has the advantage of being able to store dynamic and sparse data efficiently [20]. However, when it comes to attribute-centered query, the most frequent query mode in translational research, EAV model is less efficient than the conventional ER model. Therefore, we introduced EAV-ER mixed model, which combines advantages of the above two models, to construct the clinic-omics database. EAV tables are designed for dynamic and sparse data while relatively stable data are stored in conventional tables. 

**Figure 1:**
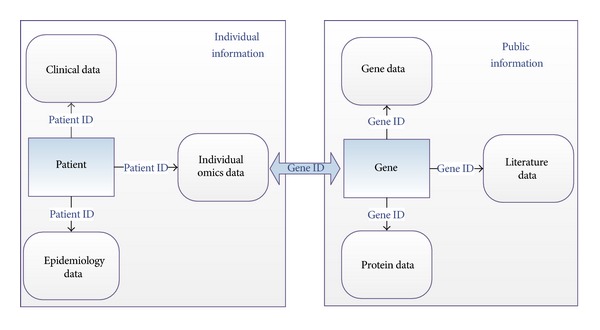
Data Relationships of the Clinic-omics Database.

Data relationships in the clinic-omics database are illustrated as Figure 1. Individual information is organized using Patient ID, while public information is organized using Gene ID. Individual information connects with public information by Gene ID of individual omics data.

### 2.3. Design of a Web Portal

**Figure 2:**
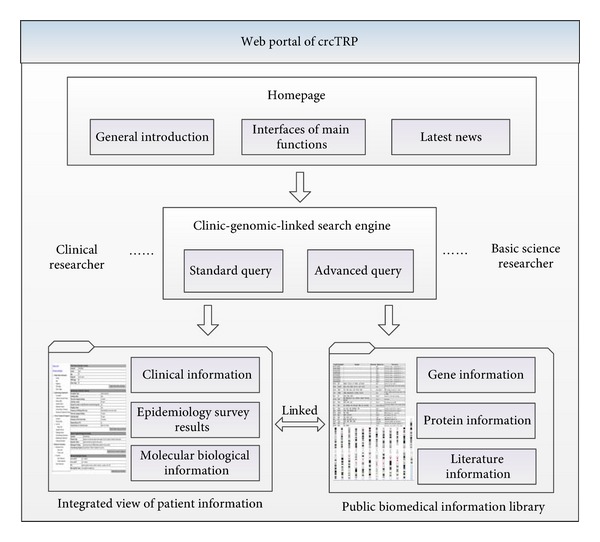
Framework of crcTRP's Web portal.

Sharing comprehensive biomedical information between clinical physicians and basic researchers does not mean providing data access to them simply. Reasonable information distribution layout should be taken into consideration for researchers to find their expected data quickly. We designed a web portal with framework shown in Figure 2 to satisfy this requirement. 

 Homepage gives an overview of the web portal and links to news reporting the latest research progress in colorectal cancer translational research. The information query engine of crcTRP's web portal consists of standard query and advanced query, to satisfy different query requirements. Besides, since different researchers may focus on different information items, query results are designed to be configurable. Researchers can download data they interested in for future use. Integrated view of patient information provides a summary view of the selected patient's whole information including clinical information, epidemiology survey results and personalized molecular biological information. Public biomedical information library offers researchers with systematic knowledge of colorectal cancer. Information in the library can be accessed through two different ways. One is clicking a certain gene in the gene list. The other is clicking a certain gene in the chromosome map, which depicts the distribution of genes highly relative with colorectal cancer in all chromosomes. Genes in the chromosome map are extracted from OMIM using method described in Section 2.1.

### 2.4. Clinic-Genomic Information Mapping Based on UMLS

**Figure 3:**
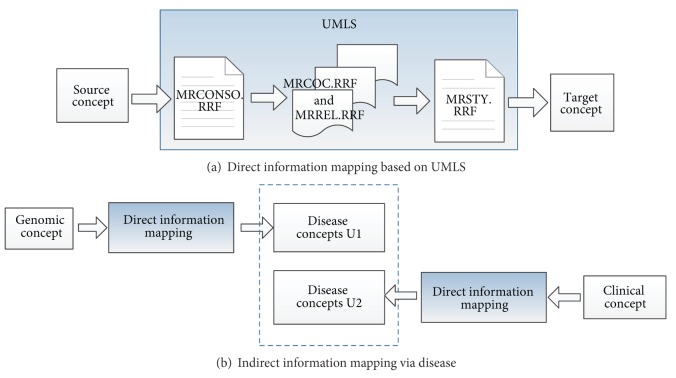
Clinic-genomic information mapping based on UMLS.

Relations between clinical data and genomic data may build a bridge between clinical practice and basic research, which is one of the key tasks of translational bioinformatics [21]. Various kinds of information acquired using the multisource biomedical information solution can be used for the potential clinic-genomic relation mining. The UMLS, or Unified Medical Language System, is a set of files and software that brings together many health and biomedical vocabularies and standards. UMLS covers a wealth of biomedical concepts from both clinical domains and molecular biology domains. Various relations among these concepts are also recorded in UMLS, laying foundation for us to implement the clinic-genomic information mapping. Installing UMLS locally will yield a series of RRF (Rich Release Format) files, including MRCONSO.RRF, MRREL.RRF, MRCOC.RRF, and MRSTY.RRF. MRCONSO.RRF lists out all concepts; MRREL.RRF contains information about the relationship between two concepts; MRCOC.RRF records cooccurring concepts; and MRSTY.RRF contains the semantic information on the concepts [22]. Two methods, namely, direct information mapping and indirect information mapping via disease, were proposed for information mapping based on UMLS. Mapping methods are shown in Figure 3.

Figure 3(a) illustrates direct information mapping method. First, search MRCONSO.RRF with source concept to get CUI (Concept Unique Identifier) of the source concept. Second, search MRREL.RRF and MRCOC.RRF to obtain concepts related with source concept using obtained CUI. Finally, search MRSTY.RRF file to obtain the semantic type of concepts found in the second step and pick out concepts with the desired semantic type. To this end, source concept is mapped to the picked out concepts. This method can be used for various kinds of information mapping. As for clinic-genomic information mapping, genomic concepts used here, called G, are genes extracted from OMIM using method described in Section 2.1. Clinical concepts used here, called C, are clinical items selected from clinical information acquired also using method described in Section 2.1. Since the clinical information we collected has been coded using SNOMED CT, concepts in C are lots of SNOMED CT codes.

Two kinds of direct information mapping, mapping from genomic to clinic or in turn, are distinguished from each other by mapping direction. When source concept is genomic concept from G, target concepts of direct information mapping aslo included in C are mapped to the genomic concept. Similarly, when source concept is clinical concept from C, target concepts of direct information mapping also included in G are mapped to the clinical concept.

Genomic information reflects disease from the micro side, indicating the mechanism of disease. Meanwhile, Clinical information reflects disease from the macro side, recording symptoms or manifestations of disease. Therefore, disease concepts can be used to relate clinical data with genomic data. The procedure of indirect mapping via disease concepts is shown in Figure 3(b). First, disease concepts mapped to genomic concept, named as U1, are obtained using direct mapping method by picking out concepts with disease semantic type in the last step of direct information mapping. Second, disease concepts mapped to clinical concept, named as U2, are also obtained using the same method. Then, if a disease concept related with colorectal cancer presents in both U1 and U2, the genomic concept is thought to be mapped to the clinical concept.

## 3. Results and Discussions

A translational research platform for colorectal cancer, named crcTRP, was developed and lots of clinic-genomic relations were found out based on information provided by the platform. Several kinds of biomedical data were collected using a multisource biomedical information collection solution and integrated in a clinic-omics database. crcTRP serves for colorectal cancer translational research by sharing these data through a unified web portal. Those clinic-genomic relationships were out of the preliminary exploration of the capabilities of the platform and have been used to relate integrated view of patient's comprehensive information with public biomedical information library on the web portal of crcTRP, which enriched crcTRP in return. Much more potential research fruits are expected to be gained by users of crcTRP. 

### 3.1. crcTRP

crcTRP consists of three parts as follows.

#### 3.1.1. A Multisource Biomedical Information Collection Solution

Based on methods described in Section 2.1, the information collection solution is able to collect clinical data, epidemiology data, individual omics data as well as public omics data. We have collected comprehensive data of more than 150 patients with colorectal cancer at present. Besides, a total of 384 data items related with clinical and epidemiological information have been coded using SNOMED CT. Epidemiology information collected using our epidemiology questionnaire system covers patients' dietary habits, personal medical history, family disease history, and other risk factors of cancer. A total of 62 genes related with colorectal cancer were extracted from OMIM, including BRAF [23] and APC [24], two important biomarkers of colorectal cancer. In addition, 54 proteins and 2006 articles related to colorectal cancer were extracted from public biomedical database by using the 62 genes.

#### 3.1.2. A Clinic-Omics Database

A database with the capability of integrating clinical and omics data was constructed. Data acquired using the multisource information collection solution were already stored in this database, which illustrated the integration capabilities of the database well. With EAV-ER model, the database is flexible enough to adapt to the dynamic and sparse nature of biomedical data while guaranteeing the efficiency of attribute-centered query. For example, biomaterial and biomarker are entities and a biomaterial may have several biomarkers. We constructed two tables named “Biomaterial” and “Gene_Biomarker” to stand for biomaterial and biomarker, respectively. Obviously, “Biomaterial” and “Gene_Biomarker” form a typical ER model. Gene sequence and mutation status are two common attributes of biomarker. In table “Gene_Biomarker,” column “Sequence” and “Is_Mutation” are used to record the corresponding attributes according to the ER mode, which is beneficial to the attributed-centered query. However, with rapid development of molecular biotechnology, new attributes will be found undoubtedly. In order to record those new attributes without modifying database architecture, we designed another two tables, “New_Gene_Biomarker” and “New_Gene_Biomarker_Attribute.” “New_Gene_Biomarker” was designed to be an EAV table, including three columns to record entity, attribute and value respectively. New attribute can be expressed via referring the primary key of table “New_Gene_Biomarker_Attribute” by “New_Gene_Biomarker.” This way, both ER model and EAV model are employed, forming the mixed EAV-ER model, to achieve the best performance as much as possible. The clinic-omics database was implemented in SQL Server 2008 with a total of 174 data tables, of which 19 for clinical data, 50 for epidemiology data, 99 for individual omics data, and 6 for public biomedical information. 

#### 3.1.3. A Web Portal

**Figure 4:**
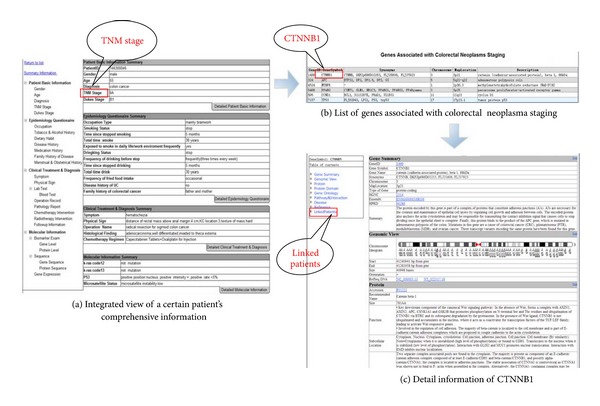
Representative screenshots of crcTRP web portal.

The web portal was developed using ASP.NET in Microsoft Visual Studio 2010 and has been deployed in IIS server. It can be accessed via http://60.191.25.26:8088/. This web portal is useful for both clinical researchers and molecular biologists. For clinical researchers, they can learn the molecular mechanism of colorectal cancer, which may lead to better understanding about the diagnosis, therapy, and prognosis of colorectal cancer. For molecular biologists, they can study the function and phenotype of genes and molecular pathways.  Figure 4 shows some screenshots of the web portal (red annotations and blue arrows will be explained later).  Figure 4(a) is the integrated view of a certain patient's comprehensive information. Information covering by the integrated view includes patient basic information, epidemiology information, clinical information, and molecular information. Tree view on the left can bring researchers to the right part quickly.  Figure 4(b) shows a gene list, which is one of the entrances of public information library. Clicking any one record on the list will come to the detail information of the selected gene, as shown in Figure 4(c). Detailed information integrates gene, protein, literature and other information, giving a systematic knowledge to researchers.

### 3.2. A Set of Clinic-Genomic Relations about Colorectal Cancer

A total of 50 clinical information items and 62 genes selected from the clinic-omics database, forming C and G mentioned in Section 2.4, were used for the information mapping. Using direct mapping method, we detected 170 relations between clinical data and genomic data, 142 from genomic to clinic and 28 from clinic to genomic. Using indirect mapping method, we detected 611 relations. Combining all these mapping results, we generated a total of 781 candidate clinic-genomic relations for colorectal cancer, without consideration about the overlap between relations generated from direct method and those from indirect method.

Appearing in the same article, a clinical concept and a genomic concept can be considered as relating to each other in some ways. Based on this idea, we proposed a relation validation method to select more closely linked relations by searching published articles. PubMed [18] is the most widely used biomedical literature retrieval system. Most articles in PubMed are assigned to Medical Subject Headings (MeSH) [25], a controlled vocabulary thesaurus of indexing terms arranged in a hierarchy, which provides a consistent way to find citations, even when authors use different terms for the same concept [26]. Furthermore, using MeSH has been shown to improve the efficiency of search, which means retrieving fewer irrelevant citations [26].

**Figure 5:**
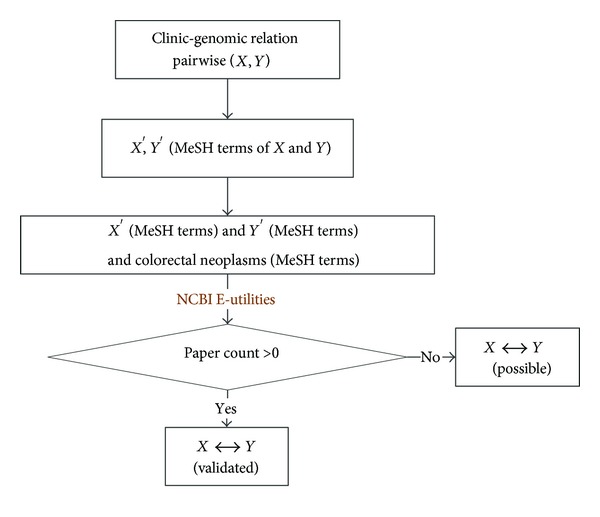
Procedure of PubMed based relation validation.

Taking the above factors into consideration, specific scheme of our relation validation method is to search PubMed online database based on MeSH ontology. The validation procedure is shown in Figure 5. Given that two concepts, *X* and *Y*, form a relation pairwise (*X*, *Y*). To validate this relation, MeSH terms of *X* and *Y*, denoted as *X*′ and *Y*′, respectively, are extracted by searching MRCONSO.RRF from UMLS using CUI of *X* and *Y*. Then, an online literature search is performed through NCBI E-utilities, a program provided by PubMed. The search keywords are defined as “*X*′[MeSH Terms] AND *Y*′[MeSH Terms] AND Colorectal neoplasms [MeSH Terms].” Term of “Colorectal neoplasms” guarantees the hit papers are related with colorectal cancer. If there is at least one article meeting the searching condition, the relation pairwise (*X*, *Y*) is thought to have passed the validation. While those relations having not passed this validation are treated as less closely linked relations at current cognitive level of human.

After validation, a total of 249 relations remained. Representative relations are presented in Table 1. Clinical data items listed in Table 1 cover most types of the focused clinical information related to treatments for colorectal cancer. These types of clinical information include symptoms, blood test results, pathology states, and diseases highly related with colorectal cancer.

We have successfully utilized these relations on crcTRP to annotate the collected clinical and omics dataset. Noticing the bold text in Table 1, clinical data item “Colorectal Neoplasm Staging” is related with six genes, including APC, CCND1, CTNNB1, MTHFR, PPARG, and TP53. Clicking the “TNM staging,” another expression of “Colorectal Neoplasm Staging,” on Figure 4(a), browser will jump to the gene list page, which lists the six genes associated with “Colorectal Neoplasm Staging,” shown as Figure 4(b). CTNNB1, the first record in the gene list, has been proved to play roles in colorectal cancer [27]. Specifically, tumor CTNNB1 status has substantial modifying effects on the beneficial prognostic role of postdiagnosis physical activity [27]. Clicking CTNNB1 on the gene list will bring out the detailed information of CTNNB1, shown as Figure 4(c). Meanwhile, IDs of patients who have the clinical items related to gene CTNNB1 are listed in the “Linked Patient” part of the gene detail information page. In this way, both clinical researchers and molecular biologists may benefit from these relations. On one hand, clinical researchers may gain a clearer understanding of the mechanism of disease by viewing biology molecular information related with a certain clinical item. On the other hand, molecular biologists may discover new roles of genes from these relations or get new ideas about next research projects through patient information related with a certain gene.

More work can be done to make better meaningful use of these relations. For example, quantifying these relations and then visualizing the quantified relations using various visualization means will offer researchers with a more intuitive understanding of these relations, which may lead to another discovery.

## 4. Conclusions

Colorectal cancer is the fifth leading cause of cancer mortality in China. Integrating existing clinical, genomic, and proteomic information and sharing these information through a unified platform will expedite the transformation of basic research results into clinical practice, and therefore promote the development of innovative treatment strategies. The main contributions of our research are concluded as follows.

First, we developed a platform named crcTRP serving for colorectal cancer translational research. crcTRP is the first platform offering various biomedical data to researchers working for colorectal cancer translational research through a unified web portal.

Second, we brought out a set of clinic-genomic relations based on crcTRP and then put these relations into use on crcTRP to serve for translational research. Not only these relations themselves are very significant for bridging clinical data with genomic data, but also the idea of how to take advantage of crcTRP is worth learning.

We view crcTRP as a good starting point. Much more work remains to be done to optimize crcTRP or take advantages of crcTRP. For example, data analysis tools can be developed and published on the web portal of crcTRP for online use. Moreover, data provided by crcTRP are excellent resource for data mining and data analysis, especially for relation mining owing to the abundant data types. At present, we are conducting a research about clinic-genomic relation mining based on large repositories of gene expression data. Clinical data provided by crcTRP aid us greatly in this ongoing research.

## Figures and Tables

**Table 1:** Representative clinic-genomic relations about colorectal cancer.

Clinical data items	Genes
Abdominal pain	FOS, HFE, NRAS, TP53
Blood glucose	BAX, CCND1, CTNNB1, FGFR3, FOS, PPARG, SRG, TLR2, TP53
Carcinoembryonic antigen	APC, BAX, CCND1, CEACAM5, CEACAM7, CEACAM1, CTNNB1, DCC, EGFR, ERBB2, MLH1, MSH2, PSG2, SRC, TLR2, TP53
CA19-9 antigen	CTNNB1, NRAS, TP53, SRC
**Colorectal neoplasms staging**	**APC, CCND1, CTNNB1, MTHFR, PPARG, TP53**
Crohn's disease	APC, BAX, CCND1, CTNNB1, DCC, MTHFR, NRAS, PPARG, TLR2, TP53
Diabetes mellitus	APC, BAX, CCND1, CHEK2, CTNNB1, DCC, FGFR3, MTHFR, NRAS, PPARG, PTPN12, SRC, TLR2, TP53
Dyspepsia	PPARG
Intestinal obstruction	APC, CCND1, MSH2, PPARG, TLR2
Lymphatic metastases	MCC, TP53, APC, BAX, CCND1, CTNNB1
